# Evaluation of Fe-Mg Binary Oxide for As (III) Adsorption—Synthesis, Characterization and Kinetic Modelling

**DOI:** 10.3390/nano11030805

**Published:** 2021-03-21

**Authors:** Saif Ullah Khan, Rumman Zaidi, Feroz Shaik, Izharul Haq Farooqi, Ameer Azam, Hatem Abuhimd, Faheem Ahmed

**Affiliations:** 1 Department of Civil Engineering, Zakir Husain College of Engineering & Technology, Aligarh Muslim University, Aligarh 202002, India; farooqi_izhar@yahoo.com; 2 Department of Applied Physics, Zakir Husain College of Engineering & Technology, Aligarh Muslim University, Aligarh 202002, India; rumman.zaidi11@gmail.com (R.Z.); azam2288@gmail.com (A.A.); 3 Department of Mechanical Engineering, Prince Mohammad Bin Fahd University, P.O. Box 1664, Al Khobar 34754, Saudi Arabia; 4 National Nanotechnology Center, King Abdulaziz City for Science and Technology, P.O. Box 6086, Riyadh 11442, Saudi Arabia; habuhimd@kacst.edu.sa; 5 Department of Physics, College of Science, King Faisal University, P.O. Box 400, Hofuf, Al-Ahsa 31982, Saudi Arabia; faheem030@gmail.com

**Keywords:** arsenic contamination, nano-particle synthesis, binary metal oxide, co-existing ions, adsorption mechanism

## Abstract

Nanotechnology has received much attention in treating contaminated waters. In the present study, a facile co-precipitation method was employed to synthesize a novel iron and magnesium based binary metal oxide using a stoichiometrically fixed amount of FeNO_3_·9H_2_O and MgNO_3_·6H_2_O in a proportion of molar concentration 1:1 and was later evaluated in removing As (III) from contaminated waters. Characterization of the prepared nanomaterial was done using X-ray diffraction (XRD), scanning electron microscopy (SEM), Energy Dispersive X-ray Analysis (EDAX) and ultraviolet–visible spectrophotometry (UV-VIS). Experimental studies on batch scale were carried out, examining the effect of varying initial concentrations of metal, adsorbent dosage, application time and initial pH on removal efficiency. Arsenic removal increased on increasing adsorbent dosage (0.1–1 g/L) but trend reversed on increasing initial arsenic concentration attaining q_max_ of 263.20 mg/g. Adsorption was quite efficient in pH range 4–8. Freundlich fitted better for adsorption isotherm along with following Pseudo-2nd order kinetics. The reusability and effect of co-existing ions on arsenic adsorption, namely SO_4_^2−^, CO_3_^2−^ and PO_4_^3−^ were also explored with reusability in 1st and 2nd cycles attained adsorptive removal up to 77% and 64% respectively. The prepared nano-adsorbent showed promising results in terms of high arsenic uptake (q_max_ of 263.20 mg/g) along with facile and cost-effective synthesis. Thus, the co-precipitation technique used in this work is a simple one step procedure without any use of any precursor as compared to most of the other procedures used for synthesis.

## 1. Introduction

Arsenic is a well known carcinogen present in water supplies all around the globe. It is prioritized as one of the top 20 hazardous substance by Agency for Toxic Substances and Disease Registry [1]. It has been reported recently in countries like China, India, Taiwan, Poland, Argentina, Japan, New Zealand, Mexico, Hungary, Canada and USA [2]. Regulatory bodies like the World Health Organization (WHO) has a directed permissible limit of arsenic as 10 ppb in drinking water [3]. Arsenic exists mostly in inorganic form besides organic as well; however the predominant forms of inorganic arsenic found in surface and ground water are arsenite and arsenate [4]. Among these two, arsenite [As (III)] is considered more toxic, as well as mobile, than arsenate, thereby drawing attention worldwide [5]. Arsenite comprises of a neutral state of arsenic species like HAsO_3_^0^ as compared to arsenate species like (H_2_AsO_4_^−^, HAsO_4_^2−^), as a result of which adsorption and other techniques generally prove less effective in removing As (III) [5]. Ingesting water that contains arsenic beyond the permissible limit for longer durations causes ill health effects, mainly cancer of skin, kidneys and lungs beside others [1].

With the technological advancements, researchers with a focus on separation techniques have used various methods for arsenic removal, including ion exchange, chemical reduction, reverse osmosis, electrochemical means, modified coagulation/filtration and adsorption, which are few among so many [6]. But these conventional approaches have one or the other drawbacks: low efficiency, requiring chemicals, huge energy consumption and extensive sludge generation [7]. With such constraints, adsorption based approaches have a wider acceptability due to ease of operation, higher efficiency and cost effectiveness [8,9].

Ongoing research on nano-sized adsorbents has attracted a lot of attention as these possess a large surface area offering a large number of active sites to bind upon [10]. The smaller the size, the larger the surface area available, thus enhancing the adsorption over their surface [11]. But still, the separation and regenerative ability of nanoparticles is a concern [12]. Several adsorbents have been reported to adsorb arsenic effectively with a focus shifting towards metal oxide based sorbents such as titanium [13], aluminum [14], zirconium [15], iron [16], and so forth. Among these, iron based sorbents were extensively studied due to greater affinity, cost effectiveness and environmental suitability [17].

In recent times, the development of adsorbents based on two or more metal oxides has attracted the attention of researchers worldwide, as binary oxide not only procures the characteristics of the parent compound but also displays synergistic effects [18]. This synergistic effect is evident from the enhanced adsorption capability in many recent studies [19,20]. For example, Fe–Mn binary oxides were found to be successful in removing arsenic ions from groundwater [21]. Another group of researchers, (An and Zhao. 2012) prepared a new class of Fe-Mn binary oxide with water soluble starch as a stabilizer for the adsorptive removal of both arsenite and arsenate [22]. Similarly, (Ren et al. 2011) prepared a Fe-Zr binary oxide that has high adsorption capacity for arsenic [3]. Another research work on synthesizing a Fe-Ce binary oxide for the possible adsorption of arsenic was explored [23]. In another study, (Khan et al. 2016) prepared a novel iron and copper based binary oxide nanomaterial for the efficient sorption of Cr (VI) [24]. Moreover, aiming at multiple contaminants, a Fe-Ce binary magnetic mesoporous metal oxide adsorbent was synthesized exhibiting simultaneous removal of Cr (VI) and As (III) [25]. The use of magnesium as an adsorbent has also been used in the past for containment of heavy metal discharge [16,26]. Even the triple metal composite of Fe-Mg-La has been successfully synthesized with magnesium as an important component for the adsorption of contaminants such as fluoride [27].

Therefore, coming up with developing novel, effective and reliable alternative, Fe (III)-Mg (II) binary oxide nano-adsorbent was prepared. A co-precipitation technique was adopted as it is a simple one step procedure without any use of any precursor as compared to most of the other procedures used for synthesis. Thus, our aim is to: (1) propose a simple method of co-precipitation to prepare this binary metal oxide nano-adsorbent at room temperature; (ii) to characterize the prepared adsorbent nanomaterial; (iii) to evaluate the As (III) removal efficiency by prepared nanomaterial in terms of adsorption capacity and study the adsorption kinetics as well as factors affecting it.

## 2. Materials and Methods

### 2.1. Chemicals and Instrumentation

Synthetic solutions of known arsenic concentration [As (III)] were prepared by dissolving NaAsO_2_ (Sigma-Aldrich, Saint louis, MI, USA) in deionized water. The beakers were air tight and covered with silver foil along with centrifuge tubes to avoid oxidation reactions resulting in conversion to As (V). Batch studies were carried out to investigate the suitability of prepared adsorbent in uptaking As (III). Arsenic concentration was determined throughout the study using Inductively coupled plasma mass spectrometry (ICP-MS) (PerkinElmer NexION 2000).

Synthesis of nanomaterial was carried out using ferric nitrate nonahydrate (FeNO_3_·9H_2_O) (Sigma-Aldrich), magnesium nitrate hexahydrate (MgNO_3_·6H_2_O) (Sigma-Aldrich). For X-ray diffraction (XRD), Rigaku Miniflex X ray diffractometre (HyPix-400 MF) with Cu-Kα rays was used for characterizing the prepared adsorbent. Fourier Transform Infrared Spectroscopy (FT-IR) was carried out by spectrophotometer (Perkin Elmer Spectrum 100). Scanning electron microscopy (SEM) for studying the morphology of the prepared material was done by electron microscope (SEM- JEOL JSM 6510LV). Optical properties were studied through ultraviolet–visible spectrophotometry (UV-VIS) spectrophotometer.

### 2.2. Nano-Adsorbent Synthesis

The binary Fe (III)-Mg (II) nanomaterial was synthesized at room temperature using iron and magnesium salts. In a typical synthesis method, stoichiometrically, a fixed amount of FeNO_3_·9H_2_O and MgNO_3_·6H_2_O were mixed in the proportion of molar concentration 1:1 dissolved in double distilled water. The sample was agitated for 2 h. Furthermore, drops of ammonia solutions (3 mol/L) were added to raise the pH to about 8, thus initiating precipitation of Fe (III)-Mg (II) oxides. The suspension formed was constantly stirred for 45 min, matured at room temperature for 24 h before washing it by de-ionized water. The dried powder obtained was calcined at 500 °C for 2 h and grinded in a mortar to obtain a fine powder. The magnetic properties, particle size and crystallinity of magnetite and magnesium oxide nanoparticles are very sensitive to the annealing temperature [28]. Annealing temperature was chosen by carefully reviewing the literature to obtain the final crystalline phases [29,30]. The resultant suspension was filtered and dried at 80 °C for 24 h. Finally, the material was finely ground and crushed to obtain the powdered form.

## 3. Results

### 3.1. Characterization by Structural Properties: (XRD)

**Figure 1:**
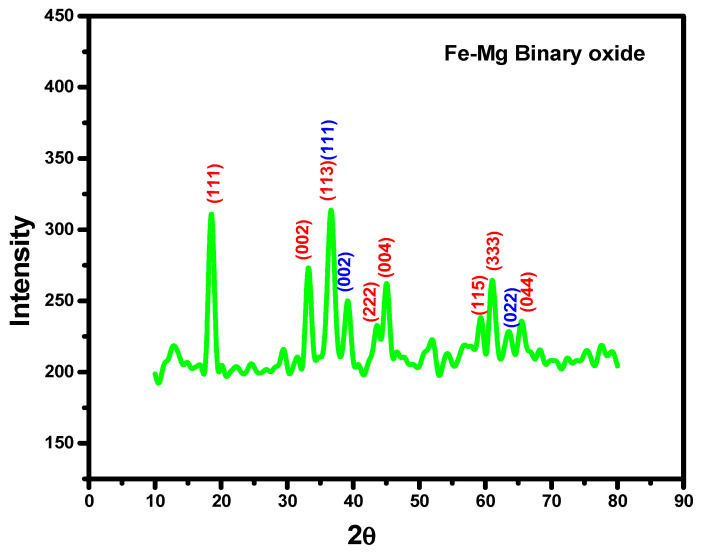
X-ray diffraction (XRD) pattern of Fe-Mg binary oxide nanoparticles.

X-ray diffraction (XRD) patterns were recorded in a Rigaku X-ray Diffractometer equipped with a graphite monochromator using CuKα radiation, 40 kV and 40 mA in the 2θ range 10–80°. X’pert highscore plus software of version 3.0 was used to identify and match the peaks. The X-ray diffraction pattern of the prepared Fe_3_O_4_-MgO binary metal oxide in 1:1 prepared by a simple, low cost co-precipitation technique is shown in Figure 1. The peak positions in the observed XRD pattern were well aligned with the literature data of both the metal oxides. The sharp and relatively strong intensive diffraction peaks reflect the highly crystalline nature of the samples. In the XRD pattern produced, all the peaks noted were attributed to either MgO or Fe_3_O_4_ facets, demonstrating that the diffraction pattern acquired is well compatible with the cubic lattice for both metal oxides, which establishes the lack of any other phases as impurities. The prepared material XRD pattern displays the diffraction peaks for iron oxide (marked red), that are observed near the 2 theta values of 18.5°, 33.6°, 37.1°, 43.4°, 45°, 59.4°, 61.1°, 65.4° with the crystal planes of (111), (022), (113), (222), (004), (115), (333), (044) for the synthesized samples (Reference code:96-901-3201, COD code: 9002331). The major peaks for MgO were detected in the XRD pattern 37.7°, 38.8°, 44.1°and 63.5° with the crystal planes of (111), (002), (022) respectively. It is well matched with Reference code: 96-901-3201, COD code: 9013200. The XRD findings suggest that a few of the of the magnesium oxide crystal plane diffraction peaks were being overlapped with iron oxide peaks, with some significant magnesium oxide peaks appearing in the Fe_3_O_4_-MgO XRD pattern confirming the presence of MgO on the Fe_3_O_4_ surface. Therefore, the prepared material is a blend of the individual phases of binary oxide that co-exist in one substance. These results are similar to other binary oxides in previous studies [31,32,33,34]. The weight percentage of Fe_3_O_4_-MgO binary metal oxide calculated was found to be 63.4% and 36.6%, respectively.

The average crystallite size of the synthesized sample is calculated by using Debyee Scherrer’s formula given in Equation (1) [35,36].

(1) $$D=\frac{0.9*\lambda }{\beta Cos\;\theta },$$

where *D* is average particle size, *θ* is Bragg’s angle, *β* is full width half maxima (FWHM), *λ* is the wavelength of CuKα radiations.

Crystallite size was calculated from the peak that has overlapping of crystal planes (111) (113) of iron and magnesium oxides. The average crystallite size thus obtained for Fe_3_O_4_/MgO nanomaterial was found to be 14 nm.

**Figure 2:**
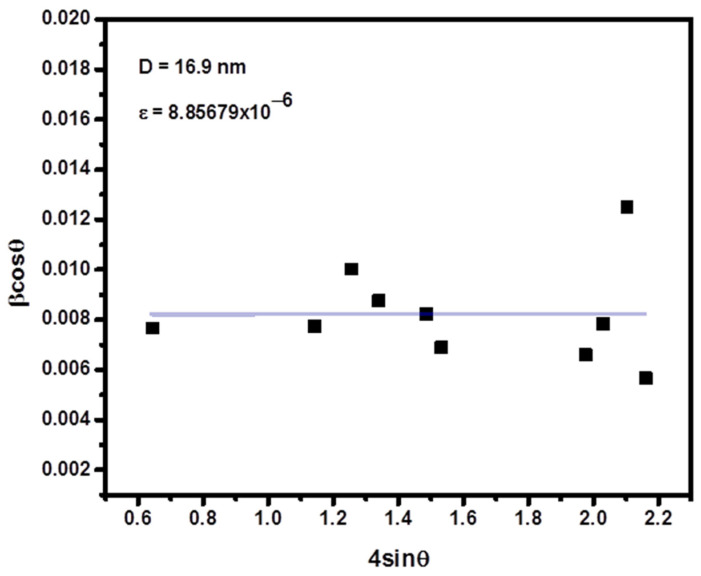
Williamson–Hall plot (W-H analysis) of Fe-Mg binary oxide nanoparticles.

For comparison and better understanding, crystallite size was also calculated by William–Hall (WH) method. The crystallite size (D) and microstrain contribute to the line broadening in X-ray diffraction. William–Hall (WH) proposed a relation between crystallite size (D) and strain (ε) induced broadening as given by Equation (2) [35,37].

(2) $$\beta Cos\;\theta =\frac{0.9\lambda }{D}+4\varepsilon Sin\;\theta ,$$

where *λ* is X-ray wavelength, 0.9 is shape, and *ε* is induced strain in crystal, *β* is the FWHM in radians. To calculate crystallite size and lattice strain of the prepared material, a graph is plotted between *βcosθ* on y-axis and 4sinθ on x-axis as shown in Figure 2. The average crystallite size and lattice strain was obtained from the slope and intercept by linear fitting of the graph respectively. A comparison between crystallite size obtained from Debye–Scherrer relation and William–Hall plots shows that crystallite size calculated from Williamson-Hall plot is slightly larger than that from Scherrer relation. This difference in the size of the crystallite is mainly due to the presence of lattice strain in the samples.

### 3.2. FTIR Studies

**Figure 3:**
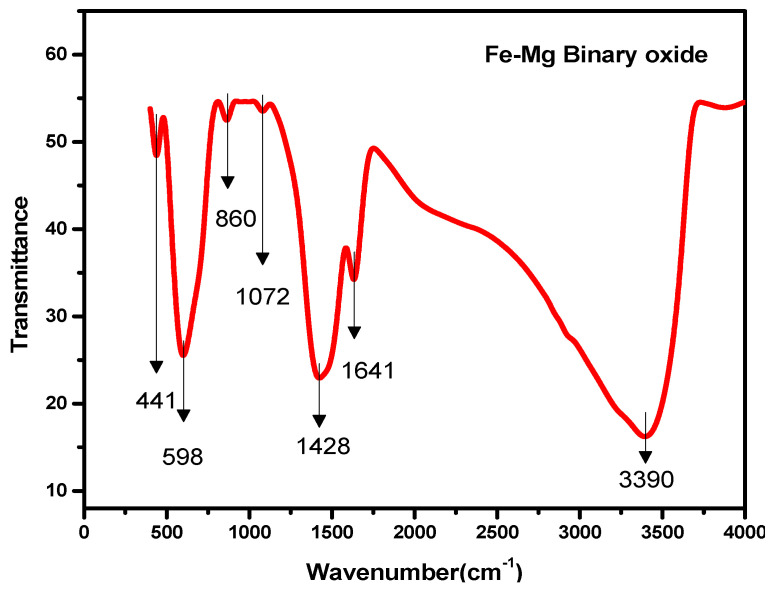
Fourier Transform Infrared Spectroscopy (FTIR) spectrum of Fe-Mg Binary Oxide Nanoparticles.

To examine the prepared nanomaterial compositions, FTIR studies were carried out via the KBr pellet method using a Nicolet FTIR spectrophotometer with a scale of 400–4000 cm^−1^ as depicted in Figure 3. The strong and broad band at 3390 cm^−1^ indicates the presence of water on the nanoparticle surface, and the vibration band at 1632 cm^−1^ and 1072 cm^−1^ can be attributed to the contribution from the OH bending vibration modes [38]. A vibration band observed at 1428 cm^−1^ can be due to the CO_3_^2−^ stretching frequency due to the adsorption of gaseous -phase CO_2_ [39,40]. It is important to note that the peak observed at 598, which can be assigned to Fe–O–Fe stretching modes, has shifted to a higher frequency from 570 when mixed oxides of Fe-Mg are formed [39,41,42,43,44]. The bands that appeared at low frequencies of 860 and 441 cm^−1^ correspond to stretching vibrations of Mg–O–Mg bonding also shows a shift towards a higher frequency range, which corresponds with previously reported work [38,45,46,47]. These results indicate the formation of nano Fe-Mg mixed oxides.

### 3.3. SEM with EDAX

**Figure 4:**
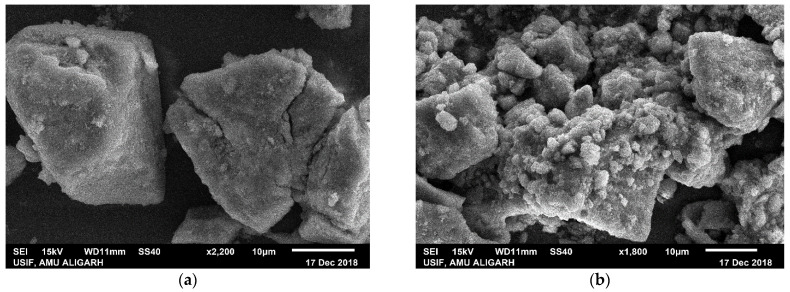

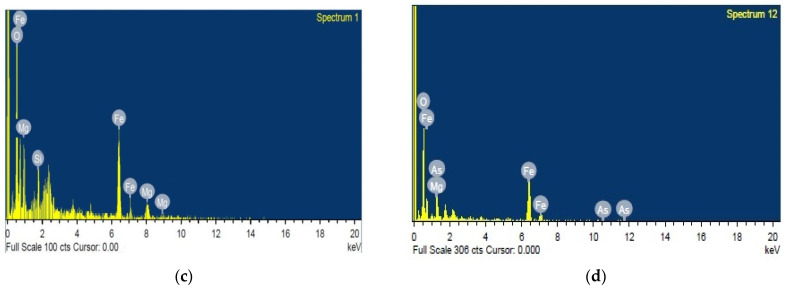
(**a**) Scanning electron microscopy (SEM) image of Fe (III)-Mg (II) binary oxide Np’s before adsorption, (**b**) SEM images after As (III) adsorption, (**c**) Spectrum from EDAX before adsorption of As (III), (**d**) Spectrum from EDAX showing presence of As (III) along with Fe and Mg after adsorption.

The morphological structures of prepared nanomaterial before and after adsorption of As (III) can be seen in Figure 4a,b using scanning electron microscopy analysis of powdered samples. Powdered sample was made to stick on the holder and was gold coated with sputter coater. SEM images before adsorption as visible in Figure 4a showing the presence of rhombohedra shaped aggregates, whereas after adsorption change in morphology showing more aggregation and presence of arsenic adsorbed over surface presumably in the form of hydroxides in Figure 4b. Spectrum graphs as shown in Figure 4c,d obtained from EDAX analysis for pre and post adsorption, confirming the adsorption of arsenic on the surface of Fe (III)-Mg (II) binary oxide nanoparticles.

### 3.4. Adsorption Experiments

Batch level studies were performed by varying As (III) concentrations and amounts of the adsorbent. The factors influencing the behavior of adsorption were explored obtaining breakthrough curves for certain fixed time durations so as to achieve equilibrium. The results obtained and plots drawn showed that prepared Fe (III)-Mg (II) binary oxide adsorbent exhibited a maximum adsorption capacity of 263.2 mg g^−1^ at pH 6. The pH of the aqueous medium had a resilient effect on adsorption process with higher efficiency in the range of 5–7. The maximum removal efficiency of 97.6% was achieved for 10 mg L^−1^ arsenic concentration at an adsorbent dose of 1 g/L and pH 6. The experimental results were repeated and found to be within ±5% error.

### 3.5. Effect of pH and Proposed Adsorption Mechanism

**Figure 5:**
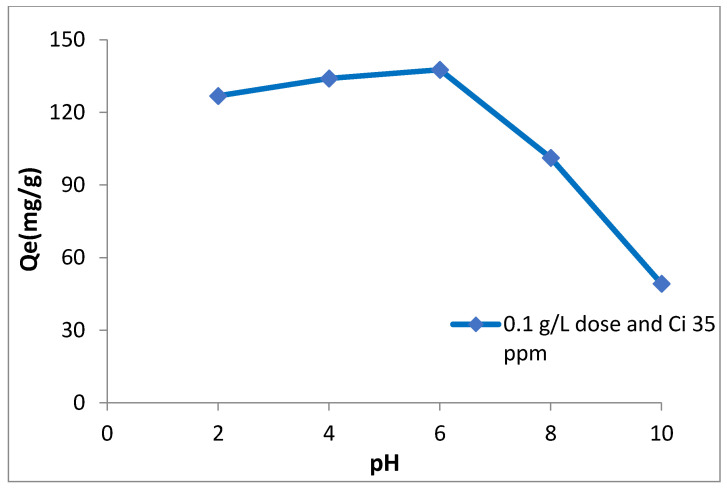
Effect of solution pH on As (III) adsorption by Fe (III)-Mg (II) binary metal oxide nanomaterial at initial As concentration of 35 mg/L and adsorbent dosage 0.1 g/L.

The adsorption of arsenic is greatly affected by the pH of the solution [4]. As is clear from Figure 5, adsorption of As (III) is higher and more effective in the range of pH 4–8. The maximum adsorption was found to be 137.6 mg/g at pH around 6. Thus we can say that adsorption is pH dependent and reduces abruptly as pH reaches 8–9. This phenomenon is found in accordance with several past studies carried out on the adsorption of arsenic on several iron oxides [3,48]. The mechanism behind As (III) removal is mainly adsorption and co-precipitation of arsenic with metal hydroxides such as iron and magnesium [1,49]. Adsorption of acid anions by oxides and hydroxides of metal usually declines as pH becomes highly basic [50]. It signifies that the prepared nanomaterial had a high reactivity at pH around 6. Moreover, at a lower pH (lower than pH of pzc), the nano-adsorbent surface will have additionally positive charges. However, arsenate comprising of H_2_AsO_4_^−^ and HAsO_4_^2−^ are the dominant negatively charged arsenic species present in the form of in the acidic medium. Thus, enhanced arsenic removal by synthesized iron magnesium binary oxide nanomaterial at lower pH shows affinity between the positively charged surface of nanomaterial and the negatively charged species of arsenic.

Both As (III) and As(V) exist as negatively charged species such as HAsO_4_^2−^, AsO_4_^3−^, H_2_AsO_3_^−^, and HAsO_3_^2−^. It is presumed that binary metal oxide nanoparticles in the aqueous solution would coordinate with hydroxyl ions and neutral water molecules. The process of the adsorption of arsenic onto the Fe_3_O_4_-MgO surface can be understood through the proposed ligand exchange mechanism. The exchange process might involve ligands, such as hydroxyl ions or neutral water molecules, existing in the metal oxide-coordinated sphere. Hence, the adsorption of arsenic might take place by the release of a hydroxyl anion or neutral water molecules from its coordinated sphere. The mechanism that might be responsible for the adsorption of the arsenic anion, that may be understood by the following equations (Equations (3)–(10)) as evident in papers by other researchers [51,52,53,54,55].

(3) $$Fe_{3}O_{4}-\mathrm{MgO}\equiv M$$

(4) $$\equiv \;M-OH+\;H_{3}AsO_{3}\to FeH_{2}AsO_{3}+H_{2}O_{\;}$$

(5) $$\equiv 2\left[MOH\right]+H_{3}AsO_{3}\to MHAsO_{3}M+2H_{2}O$$

(6) $$\equiv MOH+H_{2}AsO_{3}^{-}\to MHAsO_{3}^{-}+H_{2}O$$

(7) $$\equiv 2\left[MOH\right]+H_{2}AsO_{3}^{-}\to MHAsO_{3}M+HO^{-}+H^{+}$$

(8) $$\equiv MOH+H_{3}AsO_{4}\to MH_{2}AsO_{4}+H_{2}O$$

(9) $$\equiv MOH+H_{2}AsO_{4}^{-}+H_{2}O$$

(10) $$\equiv MOH+HAs_{4}^{2-}\to MAs_{4}^{2-}+H_{2}O.$$

### 3.6. Effect of Dosage and Initial As (III) Concentration on Kinetics

**Figure 6:**
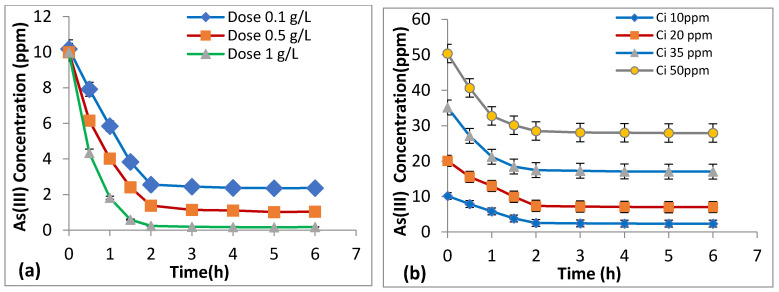
Adsorption kinetics for decrease in As (III) concentration with time at pH 6 and room temperature: (**a**) adsorption isotherm for different adsorbent dosages (0.1–1.0) g/L and initial As (III) conc. 10 mg/L w.r.t. time. (**b**) Adsorption isotherm at varying conc. (10–50) mg/L and adsorbent dose of 0.1 g/L.

The influence of dosage and initial metal concentration on As (III) adsorption with time was studied on changing the adsorbent dosages from 0.1 to 1 g/L and initial concentrations from 10–50 mg/L. Figure 6a,b gives the decrease in arsenic concentration by varying dosage and initial concentration with time. The equilibrium was achieved around 3 h in this case. From the graph (Figure 6), the sharp fall in peaks clearly indicates that the uptake capacity of the adsorbent reduces with time as the available sites for adsorption get exhausted after 3 h duration of reaction time. Adsorbent dosage greatly affects the adsorption of arsenic because of an overall increase in surface area of prepared binary oxide nanoparticles, due to higher availability of binding sites which is in accordance with several past studies [56].

### 3.7. Kinetics and Isotherm Modeling

#### Langmuir and Freundlich Isotherm 

**Figure 7:**
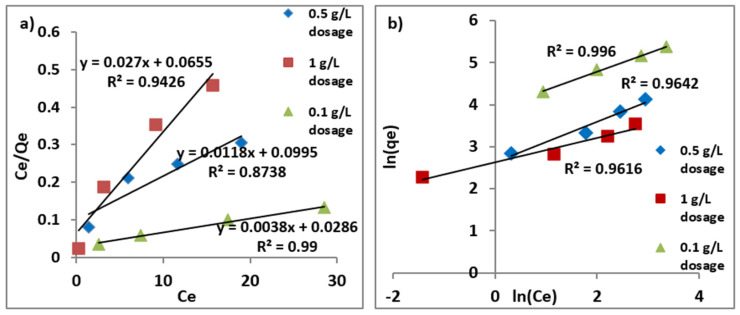
Linear models of (**a**) Langmuir adsorption isotherm at 0.1, 0.5 and 1.0 g/L adsorbent dosages, (**b**) Freundlich adsorption isotherm at 0.1, 0.5 and 1.0 g/L adsorbent dosages.

**Figure 8:**
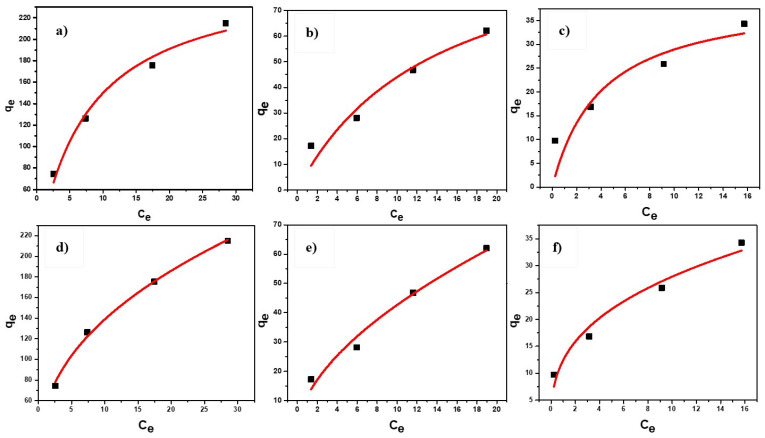
Nonlinear models of Langmuir adsorption isotherm at dosages (**a**) 0.1 g/L, (**b**) 0.5 g/L, (**c**) 1.0 g/L and Freundlich adsorption isotherm at (**d**) 0.1 g/L, (**e**) 0.5 g/L, (**f**) 1.0 g/L.

Experiments were carried out at optimized pH around 6 at room temperature to acquire data for plotting isotherms. Adsorption capacity was found to increase as more time was needed to reach the saturation point by adsorbent to adsorb maximum arsenic until the equilibrium was achieved. Both, linear and non-linear models of adsorption isotherm were plotted for the initial dosage (0.5 and 1) g/L of adsorbent and concentration (10–50) mg/L of As (III) as shown in the Figure 7 and Figure 8. Adsorption isotherms were drawn to know the best fit for both Langmuir and Freundlich models [57,58]. The linearized forms of isotherm models are as follows:

$$\ln(q_{e})=\ln\left(K_{f}\right)+\frac{1}{n}C_{e}\;\cdot (\mathrm{Freundlich}\;\mathrm{equation})$$

$$\frac{C_{e}}{q_{e}}=\frac{1}{q_{\max}b}+\frac{C_{e}}{q_{\max}}\;\cdot (\mathrm{Langmuir}\;\mathrm{equation}).$$

The non-linear isotherm models are as follows:

$$q_{e}=K_{f}C_{e}{}^{1/n}\;\cdot (\mathrm{Freundlich}\;\mathrm{equation})$$

$$q_{e}=\;\frac{q_{\max}{\mathrm{bC}}_{e}}{1+{\;\mathrm{bC}}_{e}}\;\cdot (\mathrm{Langmuir}\;\mathrm{equation}),$$

where;

q_e_ = adsorption capacity (the mass of adsorbate adsorbed over adsorbent at equilibrium), mg/g
C_e_ = the equilibrium concentration of As, mg/L
q_max_ = the maximum adsorption capacity, mg/g
K_f_ = Freundlich constant
n = Freundlich intensity factor
b = Langmuir constant

The results for varying adsorbent dosages (0.1, 0.5 and 1.0) g/L and initial As (III) concentrations (10, 20, 35 and 50) mg/L were fitted to both the linear and non-linear isotherm models as depicted in Figure 7 and Figure 8 to compute the parametric constants as obtained from these plots and were tabulated in Table 1. It may be observed that maximum adsorption capacity estimated in both cases of non-linear and linear models was found almost same as equal to 263 mg/g. Moreover, it may be concluded from the obtained plots and computation made that Freundlich model fitted better with higher value of R-square. This may be attributed to the multi-layer coverage over a heterogeneous surface with identical sites for adsorption on the surface of nanoparticles. Better fitting to Freundlich as compared to Langmuir model also implies that the adsorption of As (III) over the surface of Fe (III)-Mg (II) binary oxide was dominated by multi-layered rather than mono-layered.

### 3.8. Adsorption Kinetics

**Figure 9:**
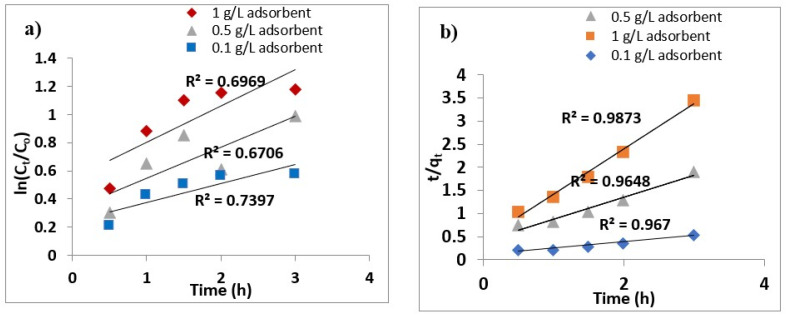
(**a**) Pseudo-first-order kinetics fitting, (**b**) pseudo second order kinetics fitting for 50 mg/L initial concentration of As (III) and adsorbent dosages 0.1, 0.5 and 1 g/L.

Studies on adsorption kinetics were carried out for both pseudo Ist order and IInd order as depicted in Figure 9a,b and was observed as to follow pseudo IInd order kinetics. Supposing that adsorption capacity is proportionate to no. of active adsorption sites in accordance with (Ho and McKay 1999) the Lagergren equation may be expressed as [59]:

$$\frac{{\mathrm{dq}}_{t}}{\mathrm{dt}}=k_{\mathrm{ads}}{\left(q_{e}-q_{t}\right)}^{2}\;q_{t}\;=\;0\;\mathrm{at}\;‘t’\;=\;0,$$

where q_t_ is the amount of As on adsorbent surface at time ‘t’, k_ads_ is the adsorption rate constant (g mg^−1^ h^−1^). On integration and rearrangement, the linearized form of pseudo second order rate may be written as follows:

$$\frac{t}{q_{t}}=\frac{1}{h}+\frac{1\;}{q_{e}}t$$

$$h=k_{\mathrm{ads}}q_{e}^{2},$$

where h is the initial adsorption rate (mg g^−1^ h^−1^). The values of K_ads_ and h were found from the slope and intercept of the graphs. The values of k_ads_ and h obtained were 0.55 g mg^−1^ h^−1^ and 2.45 mg g^−1^ h^−1^, respectively, for 0.5 g/L dosage and 2.21 g mg^−1^ h^−1^ and 2.29 mg g^−1^ h^−1^, respectively, for 1 g/L dosage adsorbent.

### 3.9. Effect of Co-Existing Ions

**Figure 10:**
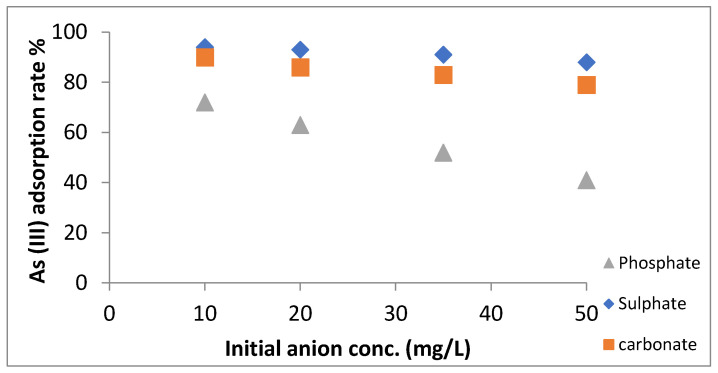
Effect of coexisting ions (PO_4_^3−^, SO_4_^2−^ and CO_3_^2−^) on As (III) adsorption.

Common anions, such as phosphate, sulphate and carbonate, may either compete or interfere with the adsorption of arsenic, as they are generally found in an aqueous medium. So their effect on adsorption was investigated at different As (III) concentrations (10–50 mg L^−1^), pH 6 and a fixed adsorbent dose of 0.5 g/L. The results are shown in Figure 10, signifying that phosphate competes and hinders adsorption of arsenic significantly, which is in accordance with past studies [60,61,62]. This may be due to the fact that both arsenate and phosphate had similar structural and chemical behavior, thereby getting adsorbed onto iron oxide forming inner sphere complex. At higher initial concentrations, the amount of arsenic adsorption decreases slightly due to inhibition as a result of coexisting ions. Furthermore, phosphate being analogue to arsenate competes well, although arsenate binds strongly onto iron hydroxides due to its larger size [63]. On contrary, carbonate and sulphate were not found to interfere in adsorption.

### 3.10. Comparison of Fe (III)-Mg (II) Binary Oxide with Other Adsorbents

Table 2 gives a comparative assessment of the maximum arsenic adsorption capacities of recently used adsorbents as reported in the literature under different experimental states. The computed q_max_ value (263.2 mg/g) of prepared binary oxide nanomaterial was found to be much higher than other reported materials for As (III) and As (V) removal.

### 3.11. Reusability of Synthesized Material

**Figure 11:**
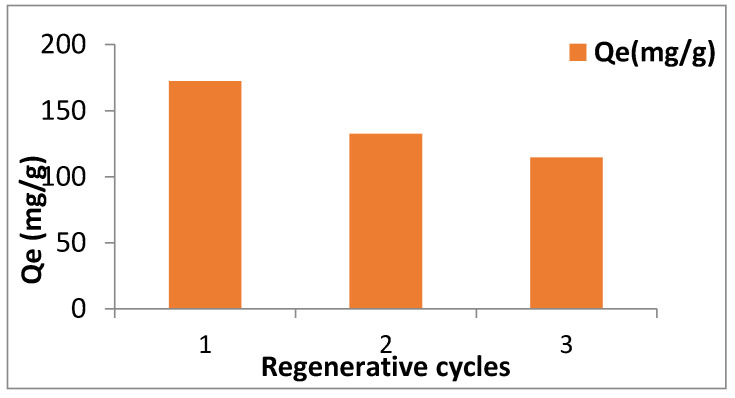
Effect on adsorption capacity of the prepared nanomaterial after regenerative cycles.

The reusability and regenerative power of synthesized binary oxide nanomaterial was also assessed by rinsing the used adsorbent with 0.1 M mol/L of NaOH solution before drying and desiccating it. Then these nanoparticles were later subjected 2 more adsorption tests for which the results were obtained as shown in Figure 11. Adsorption capacity of regenerated Fe (III)-Mg (II) oxide nanomaterial decreased with the no. of cycles as shown in Figure 11. Although, the reduction in adsorption after Ist and IInd cycle was found to be 23.2% and 13.5% respectively, signifying that percent reduction in removal efficiency was quite low after the Ist regenerative cycle.

## 4. Conclusions

A distinctive Fe (III)-Mg (II) binary metal oxide nanomaterial was synthesized by a simple co-precipitation method. The crystallite size of the prepared nanomaterial was obtained to be 14 nm as calculated using the Debye-Scherrer equation. The adsorption of As (III) on the prepared nanomaterial was controlled by both the surface charge of Fe (III)-Mg (II) and the form of arsenic species. pH was found to be an influencing factor in adsorbing arsenic as it decides the feasible pH range for adsorption to occur along deciding the dominance of particular arsenic species. The adsorption capacity of As (III) was found high in slightly acidic-neutral range and started declining as the solution shifts towards highly basic nature. These results suggest that pH needs to be optimized around slight acidic conditions; where nanomaterial is highly efficient in uptaking arsenic. Co-existing ions of carbonates and sulphates have a negligible effect on arsenic adsorption, except phosphate, which considerably reduces arsenic removal. Moreover, the prepared binary metal oxide nanomaterial could be easily regenerated to be reused. The results were conclusive in proving that prepared binary oxide might be successfully used in treating arsenite present in water supplies.

## Figures and Tables

**Table 1:** Parametric values obtained from isotherm plots for As (III) adsorption experimental runs at initial pH 6 and room temperature.

Models	Adsorbent Dosage (g/L)	Langmuir Isotherm			Freundlich Isotherm		
		*q_max_ (mg/g)*	*b*	*R* ^2^	*K_f_*	*n*	*R* ^2^
**Linear**	1	37.05	0.41221	0.9426	13.92659	2.287283	0.9616
	0.5	84.75	0.1186	0.8738	13.802	2.0492	0.9642
	0.1	263.2	0.13286	0.99	50.4761	3.460208	0.996
**Non-linear**	1	40.54	0.24854	0.819	12.42526	2.83962	0.94786
	0.5	105.15	0.07201	0.905	11.53966	1.76192	0.96806
	0.1	263.33	0.13199	0.974	52.44399	2.36656	0.99577

**Table 2:** Arsenic adsorption capacities of few recently used nano-sized adsorbents as reported.

S. No.	Adsorbents	pH	Qe (mg g^−1^)	References
(1)	Fe (III)-Mg (II) oxide NPs	6	263.2 [As (III)]	Present Study
(2)	Fe-Zr binary oxide	7	46.1 [As (V)]	[3]
(3)	GNP/Fe–Mg Binary Oxide Composite	7	103.9 [As (V)]	[16]
(4)	Nickel boride nanoparticle-coated resin	6	23.4 [As (III)]	[64]
(5)	Ce-Fe mixed oxide MWCNT	4	28.74 [As (V)]	[65]
(6)	ZnOnanorods	7	52.63 [As (V)]	[66]
(7)	Ethylenediamine modified Fe_3_O_4_ NPs	2	107 [As (V)]	[67]
(8)	Flower like porous MgO Np’s	7	252.34 [As (III)]	[68]
(9)	Granular Mn-oxide-doped Al oxide (GMAO)	7	48.52 [As (III)]	[69]
(10)	Magnetic nanoparticle-impregnated chitosan beads	6.8	35.7 [As (V)]	[70]
(11)	Superparamagnetic Mg_0.27_Fe_2.5_O_4_	7	127.4 [As (III)]	[71]
